# Supplementary material for: Myopia and Near Work: A Systematic Review and Meta-Analysis
Source: Int J Environ Res Public Health. 2023 Jan 3;20(1):875. doi: 10.3390/ijerph20010875 (PMC9820324; doi:10.3390/ijerph20010875)

Text S1. Details of search strategy

Pubmed

("myopia"[All Fields] OR "short sight"[All Fields])

AND

("near-work"[All Fields] OR "display"[All Fields] OR "user"[All Fields])

Embase

('myopia'/exp OR myopia OR 'short sight')

AND

('near work' OR (near AND ('work'/exp OR work)) OR user OR display)

Cochrane

"myopia" OR "short-sight”

AND "near-work" OR "user"

ScienceDirect

("myopia" OR"short sight")

AND

("near-work"OR "display"OR "user")

Figure S2. Quality of articles as risk of bias for each included article using the SIGN checklist and as reporting quality using the STROBE checklist

A. Risk of bias for each included article using the SIGN model

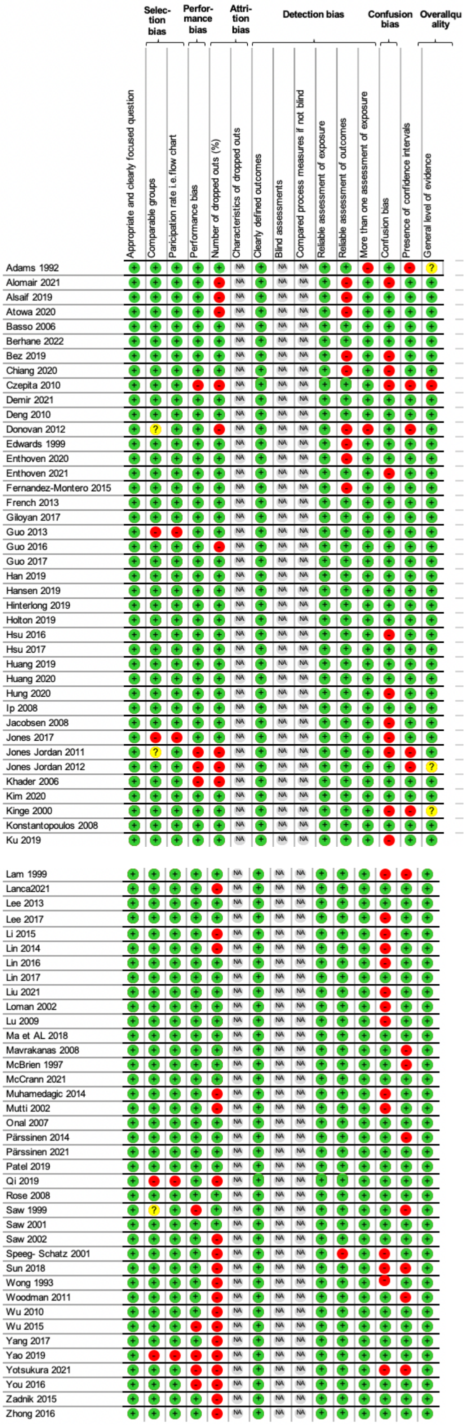

B. Risk of bias for each included article using the STROBE checklist

	Total score	Abstract Introduction	Methods	Results	Discussion
Adams 1992	65	75	50	70	100
Alomair 2021	79,0	100	80	57	100
Alsaif 2019	88,7	100	80	86	100
Atowa 2020	74,0	75	90	57	100
Basso 2006	85,7	100	100	57	100
Berhane 2022	92,0	100	90	86	100
Bez 2019	85,7	100	100	57	100
Chiang 2020	87,0	75	100	86	75
Czepita 2010	46,3	75	50	14	100
Demir 2021	90,0	100	70	100	75
Deng 2010	90,0	100	70	100	100
Donovan 2012	62,7	75	70	43	75
Edwards 1999	66,3	100	70	29	100
Enthoven 2020	81,0	100	100	43	100
Enthoven 2021	95,3	100	100	86	100
Fernandez-Montero 2015	83,3	100	80	70	100
French 2013	85,7	100	100	57	100
Gilloyan 2017	85,7	100	100	57	75
Guo 2013	85,7	100	100	57	100
Guo 2016	85,7	100	100	57	100
Guo 2017	77,7	100	90	43	100
Han 2019	95,3	100	100	86	100
Hansen 2019	81,0	100	100	43	100
Hinterlong 2019	85,7	100	100	57	100
Holton 2019	82,3	100	90	57	100
Hsu 2016	86,7	100	60	100	100
Hsu 2017	81,0	100	100	43	100
Huang 2019	95,3	100	100	86,0	100
Huang 2020	85,7	100	100	57	100
Hung 2020	81,0	100	100	43	100
Ip 2008	85,7	100	100	57	100
Jacobsen 2008	82,3	100	90	57	100
Jones 2017	85,7	100	100	57	100
Jones-Jordan 2011	46,3	75	50	14	100
Jones-Jordan 2012	51,3	100	40	14	100
Khader 2006	81,0	100	100	43	100
Kim 2020	83,3	100	80	70	100
Kinge 2000	72,7	75	100	43	100
Konstantopoulos 2008	54,7	100	50	14	100
Ku 2019	88,7	100	80	86	100
Lam 1999	74,3	100	80	43	100
Lanca 2021	88,7	100	80	86	100
Lee 2013	83,3	100	80	70	100
Lee 2017	93,3	100	80	100	100
Li 2015	77,3	75	100	57	100
Lin 2014	77,3	75	100	57	100
Lin 2016	74,3	100	80	43	100
Lin 2017	74,3	100	80	43	100
Liu 2021	77,7	100	90	43	100
Loman 2002	69,3	100	80	28	100
Lu 2009	83,7	75	90	86	100
Ma et al 2018	96,7	100	90	100	100
Mavrakanas 2000	67,7	75	100	28	75
McBrien 1997	66,0	75	80	43	100
McCrann 2021	81,7	75	100	70	100
Muhamedagic 2014	90,0	100	100	70	100
Mutti2002	77,7	100	90	43	100
Onal 2007	71,0	100	70	43	100
Pärssinen 2014	90,0	100	70	100	100
Pärssinen 2021	81,0	100	100	43	100
Patel2019	80,0	100	70	70	100
Qi 2019	83,3	100	80	70	100
Rose2008	90,0	100	80	90	100
Saw1999	76,0	100	100	28	100
Saw2001	100,0	100	100	100	100
Saw 2002	81,0	100	100	43	100
Speeg-Schatz 2001	64,3	100	50	43	100
Sun 2018	85,7	100	100	57	100
Wong 1993	61,0	100	40	43	75
Woodman 2011	81,0	100	100	43	100
Wu 2010	82,3	100	90	57	75
Wu 2015	88,7	100	80	86	100
Yang 2017	71,0	100	70	43	100
Yao 2019	90,0	100	70	100	100
Yotsukura 2021	65,7	100	40	57	100
You 2016	81,0	100	100	43	75
Zadnik 2015	82,3	100	90	57	75
Zhong 2016	74,3	100	80	43	100

Figure S3. Metaregressions on factors influencing the myopia progression per year

Variables	Coefficient (95CI)		p-value
**Population**			
Age, adults vs <18 years old	0.19	(-0.08 to 0.45)	0.15
Sex, male as reference	0.00	(-0.01 to 0.01)	0.73
Parental history of myopia	-0.13	(-0.46 to 0.20)	0.40
Educational level	0.07	(-0.23 to 0.39)	0.60
**By continents**			
Asia vs Europe	0.20	(-0.10 to 0.49)	0.17
Asia vs North America	0.25	(-0.13 to 0.63)	0.095
Asia vs Australia	0.38	(-0.13 to 0.91)	0.13
Europe vs North America	0.25	(-0.13 to 0.63)	0.18
Europe vs Australia	0.38	(-0.13 to 0.91)	0.19
North America vs Australia	0.13	(-0.47 to 0.74)	0.64
**By time**			
<2005 vs 2005-2015	0.09	(-0.24 to 0.43)	0.55
<2005 vs >2015	-0.12	(-0.44 to 0.20)	0.43
2005-2015 vs >2015	-0.21	(-0.49 to 0.06)	0.12
**Duration and kind of near work**			
Reading papers, per 10-hour/week	-0.01	(-0.04 to 0.02)	0.37
Outdoor activity, per 10-hour/week	0.05	(-0.08 to 0.19)	0.25
VDT exposure, per 10-hour/week	0.00	(-0.02 to 0.03)	0.75

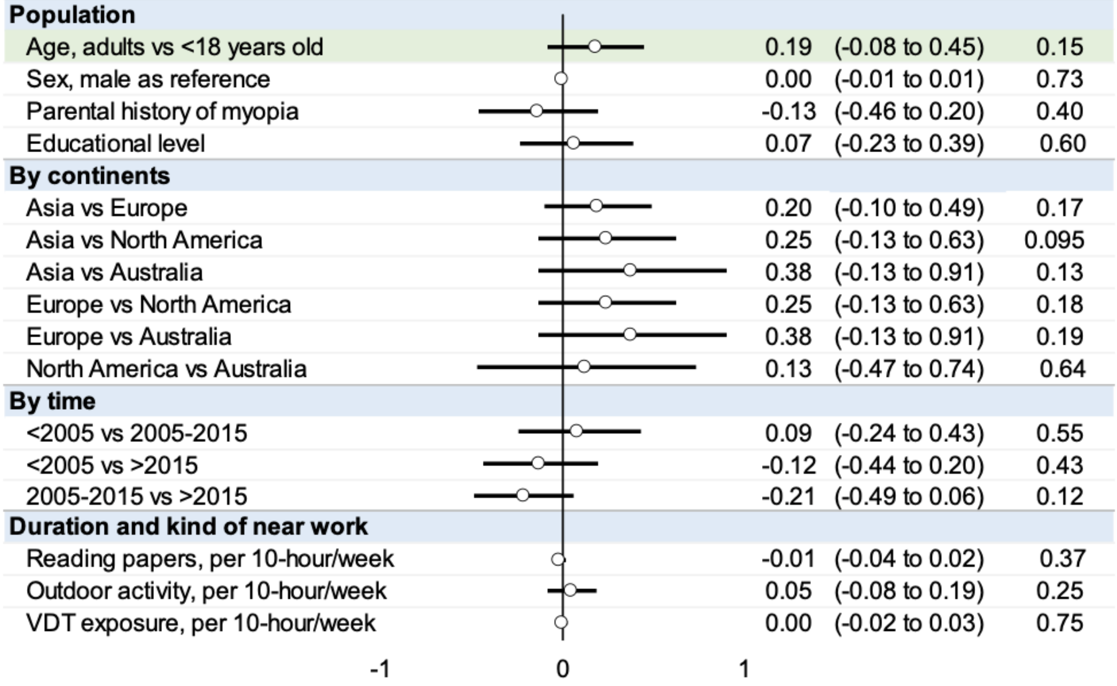

Figure S4. Metaregressions on factors influencing the odds ratio for myopia

Variables	n subgroups	Coefficient (95CI)		p-value
**Population**				
Age, adults vs <18 years old	37	0.06	(-1.11 to 1.24)	0.91
Sex, male as reference	43	0.00	(-3.00 to 3.00)	0.98
Educational level	18	0.17	(-1.14 to 1.47)	0.79
**By continents**	36			
Asia vs Europe		0.03	(-1.18 to 1.23)	0.96
**By time**	37			
<2005 vs 2005-2015		-0.35	(-2.26 to 1.56)	0.70
<2005 vs >2015		-0.03	(-1.70 to 1.60)	0.96
2005-2015 vs >2015		0.32	(-0.96 to 1.60)	0.61
**Duration and kind of near work**				
Reading papers, per 10-hour/we	12	0.00	(-0.05 to 0.05)	0.97
VDT exposure, per 10-hour/wee	14	-0.04	(-0.80 to 0.71)	0.71

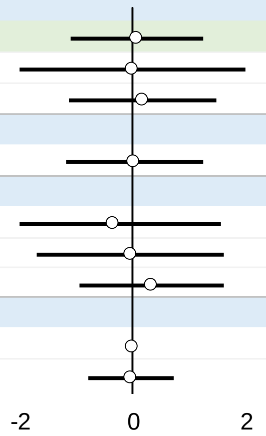

Supplement: Supplementary file 1 [file ijerph-20-00875-s001.zip › ijerph-2069783-supplementary.pdf]
